# Continuous Positive Airway Pressure Adherence for Obstructive Sleep Apnea

**DOI:** 10.5402/2011/943586

**Published:** 2011-06-30

**Authors:** Melissa L. Somers, Ed Peterson, Saurabh Sharma, Kathleen Yaremchuk

**Affiliations:** ^1^Department of Otolaryngology Head-Neck Surgery, Henry Ford Medical Group, 2799 W Grand Boulevard (K8 Clinic Building), Detroit, MI 48202, USA; ^2^Department of Biostatistics, Henry Ford Health System, Detroit, MI 48202, USA; ^3^School of Medicine, Wayne State University, Detroit, MI 48201, USA

## Abstract

*Objective.* To determine predictors of patient adherence to CPAP. *Design.* A retrospective chart review identified patients with AHI values greater than 15 who were recommended to receive CPAP. Compliance was measured at a 1-to 4-month interval and at 1 year. *Results.* There were 106 of 368 (29%) patients who received CPAP therapy that were compliant with CPAP use at 1 to 4 months. Forty-six patients (12%) were using CPAP at one year. For the male group at one year, the model demonstrated the AHI value (*P*  value = .026) as a predictor of compliance if greater than 27.3 and a significant two-way interaction between age and AHI (*P* = .023). Increased length of time from the initial visit and receiving the CPAP machine was associated with poorer compliance (*P* = .002). Those living in areas with higher incomes and with a higher percentage of non-high-school graduates were more likely to be compliant (*P* = .01 and *P* = .044). *Conclusion.* Older male patients with higher AHI values were noted to be more adherent to CPAP. Efforts should be made to try to minimize the length of time between the initial visit and the time to receive CPAP to improve compliance.

## 1. Introduction

Obstructive sleep apnea syndrome (OSAS) is present in as many as 2% of females and 4% of males in the general population. [1] It may lead to excessive daytime sleepiness with an increased incidence of automobile accidents and industrial mishaps. It is also associated with an increased risk for cardiovascular events such as stroke and myocardial infarction if untreated. Continuous positive airway pressure (CPAP) is the treatment of choice for obstructive sleep apnea syndrome (OSAS) and has been found in randomized controlled trials to be highly efficacious. However, there is a distinction between efficacy and effectiveness. Efficacy is the effect in the lab or under ideal circumstance, regardless of treatment compliance. CPAP demonstrates very good efficacy. Effectiveness is the effect in daily life, which depends on patient compliance with therapy [2]. Compliance with CPAP therapy has been found to be problematic, so this study evaluates factors that may negatively impact adherence.

CPAP is instituted following diagnosis of sleep apnea from an overnight polysomnogram. The patient returns to the sleep laboratory on another night for a repeat polysomnogram with CPAP in place. At timed intervals, pressure levels are increased until obstructive events are eliminated. Often times, patients are intolerant of CPAP as a treatment modality, and nonadherence is an issue. Many insurance companies and The Centers for Medicare & Medicaid Services (CMS) have implemented guidelines that require documentation of the patients' use of CPAP during the first month of therapy. If poor compliance is evident, recommendations are made to adjust pressure settings or refitting of the mask. If the CPAP is not being used on a consistent basis, payment from the insurance company may be terminated and the patient asked to return the equipment or assume responsibility for future costs. 

The goals of the study were to determine the length of time from initial visit to the time CPAP therapy for treatment of OSAS begins, to determine the percentage of patients that are lost to followup during the process of diagnosis to treatment of OSA, and for one year after the CPAP machine is received. An analysis was performed to determine if age, gender, race, apnea-hypopnea index (AHI), or socioeconomic status predicts patient adherence to CPAP at one month and one year.

## 2. Materials and Methods

Before the induction of this study, full approval was granted by the Henry Ford Health System Institutional Review Board. A retrospective chart review was performed to identify patients that had a polysomnogram (CPT codes 95810 and 95811) performed between January 2005 and December 2007. Clinical data was obtained from review of the sleep study results and clinical records. Parameters that were included in the data collection were patient age, sex, socioeconomic status, race, and AHI. Socioeconomic status (SES) variables were based on the United States Census results based on the area where the patient lived and included the median household income, the average household size, this size stratified by whether the people living in the house were owners or renters, and the level of education. Patients were included if they had insurance with benefit coverage that included durable medical equipment. This was done to eliminate individuals that did not obtain CPAP because of financial reasons but could be considered noncompliant. Patients were selected for the study if they had an apnea-hypopnea index greater or equal to 15 on the polysomnogram. AHI was defined as the mean number of apnea and hypopnea events per hour of sleep. Apnea was defined as the absence of airflow for 10 seconds, and hypopnea was defined as a 50% reduction in airflow for a minimum of 10 seconds per the protocol of the Sleep Center that performed the studies. 

The current clinical pathway for diagnosis of obstructive sleep apnea requires multiple visits and may contribute to the lack of followup, diagnosis, and treatment. Patients will most commonly complain to a primary car physician that they are sleepy, snore, or have bed partners who tell them that they stop breathing during their sleep. The primary care physician then refers them to a sleep specialist who will evaluate the patient to determine if a sleep study will be of benefit. CMS guidelines require the patient to be seen by a sleep specialist prior to the polysomnogram (PSG) or sleep study. A sleep study is performed to identify if the patient has OSA. The patient leaves the sleep center the next morning, and the PSG is interpreted by a sleep physician. This may take several days to evaluate all the parameters that are included in a PSG. The patient then returns to clinic for PSG results. If OSA is identified, a CPAP titration is scheduled which is a PSG with CPAP to determine optimal pressure to eliminate apneic events. A return appointment is made for the patient with the sleep physician to discuss the results and to order the CPAP machine, tubing, and mask. The delivery of equipment may take a week or two depending on the patient's availability. After one month, the patient must return to the physician to have the CPAP usage evaluated and if usage is less than required, adjustments are made to the pressures settings, mask, or equipment. 

Of the patients that received CPAP, clinical notes were reviewed to determine if the patients were compliant with CPAP at 1–4 months and at 1 year. Adequate CPAP usage was defined for our study as an average of greater than 5 hours per night for the majority of nights of the week.

The patients were categorized based on documented usage. Those that subjectively reported greater than 5 hours per night of usage and those with objectively measured hours on the CPAP therapy were considered adherent to therapy. The patients in our study that were intolerant of CPAP or were using the machine less than the recommended hours per night either by subjective or objective reporting were deemed nonadherent. Those that were lost to followup or were subjectively using their machines without documentation of time usage were considered noncompliant.

To approach the statistical analysis, we first evaluated all variables for normality. The various estimates of time between events were skewed. A logarithmic transformation on these variables brought them into normality.

We looked at the relationship between the various variables, demographic and socioeconomic using Pearson's correlation coefficients. Student's *t*-tests were used to examine differential time effects based on age or gender.

The compliance rates were conservatively estimated as the number of compliers over the number of noncompliers. Included in this latter group were all patients where we had no information. The 95% confidence intervals were calculated using the exact binomial approach.

A descriptive analysis was done on comparing the persistent compliers to the noncompliers. This is considered descriptive due to the extremely small sample size available for analysis in the non-complier group (*n* = 4). We report *P*-values here only as a guide.

We did a similar analysis on long-term compliers versus noncompliers. The variables were considered one at a time using either Student's *t*-test for continuous variables or chi-squared tests for categorical variable.

We then utilized two separate multiple variate logistic regression approaches. The first focused on predicting 1-year adherence by the various demographic variables. The focus was on evaluating possible effect modifications. A stratified approach was ultimately used to fully describe the relationships in the data. A second multiple variate logistic regression was used to evaluate the various log-transformed time variables and the socioeconomic variables characterizing the population. As this set of potential variables was large, we utilized a backward stepwise routine to identify the best set of predictors for the model.

## 3. Results

**Figure 1:**
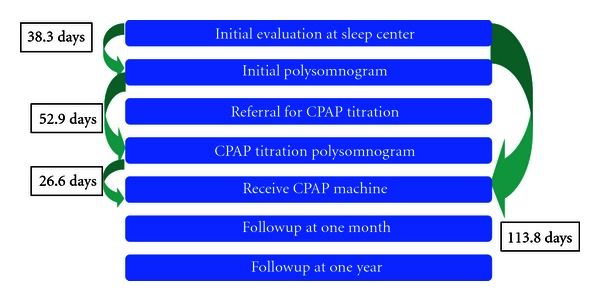
Typical course from referral for sleep apnea evaluation to receiving CPAP machine. Total length of time from initial evaluation at a sleep center to the time of receiving the CPAP machine is a mean of 113.8 days.

A total of 860 patients that had a polysomnogram were chosen for the study based on having insurance coverage for durable medical equipment that would cover costs associated with CPAP. Four hundred and fifty-six patients were noted to have an AHI value greater or equal to 15. The geometric mean time from the first appointment in the sleep center to the polysomnogram was 23.7 days (median =25.0). The geometric mean time from the polysomnogram to CPAP titration was 35.4 days (median = 41.0). The geometric mean time from CPAP titration to receiving CPAP machine was 17.3 days (median = 15.0) (Figure 1). From the initial visit at a sleep center, it takes a geometric mean time of 85.9 days to receive the CPAP machine. 

The log-transformed time period between the polysomnogram and CPAP titration was significantly correlated with the average income where subjects lived with higher incomes being associated with increased times (Pearson correlation = 0.15, *P*  value = .005). It was also the case that this time was shorter for African Americans with the geometric mean for African Americans equal to 30.2 days compared to 39.6 days for non-African American (*P* = .008). The time was also shorter for males, 33.3 days versus the females geometric mean of 43.2 days (*P* = .022). The total time interval was significantly correlated with the average household size in the area where the person lived with higher sizes being related to longer waits (correlation = 0.12, *P* = .024). There was also a longer total time interval associated with larger household sizes if you looked at homes in which the owner lived (correlation = 0.13, *P* = .011). 

**Figure 2:**
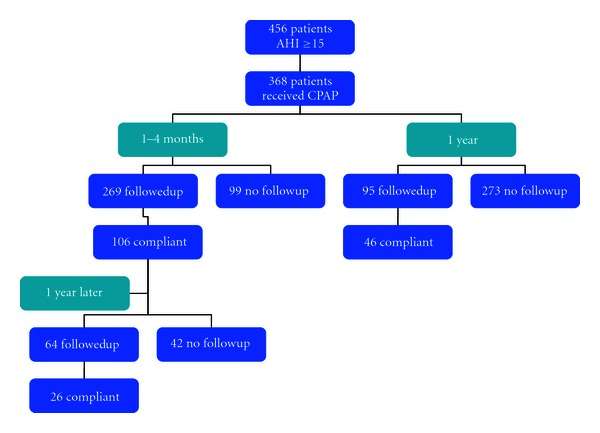
At one month after receiving CPAP machines, 106 of 368 (29%) were considered compliant with their machine usage. There were a total of 26 patients of the patients that were compliant at one month that continued to use their machine at one year (7%). Forty-six patients of the original 368 that received CPAP (13%) were using their machines at one year.

Three hundred and sixty-eight patients of the 456 patients with AHI values greater or equal to 15 received machines based on insurance claims. Of these patients, 24.3% were female and 37.2% were African American with an average AHI value of 43.7 ± 24.8. Of the 368 patients who received a machine, 98 patients came to their first follow-up appointment and were objectively noted to be adherent with CPAP. Eight showed up without their machine but stated subjectively that they were using it at least 5 hours per night. Sixty-six patients at their initial visits had CPAP machine readings which showed a use pattern less than the recommended time and were considered noncompliant. Five patients, without their machines, reported being noncompliant. Twelve patients did not tolerate the machine and were not using it. The remaining 179 subjects had no follow-up information available in the first 4 months and were considered noncompliant. This gave us a compliance rate of 28.8% with a 95% confidence interval of 24.2–33.7% (Figure 2).

Of the 106 patients that were compliant either by subjective or objective measures at the initial followup, 64 were followedup at 1 year or longer with either their primary care physician or the sleep center. Of these, twenty two had objective evidence and 4 reported subjective evidence of use of their CPAP for 5 hours per night or longer at one year. There was information available on 4 patients, 3 using CPAP less than 5 hours per night and one who had discontinued use to compare to the 26 compliant patients. The remaining patients had no information available and were classified as noncompliant. The rate of compliance at 1 to 4 months and 1 year was 7.1% (95% confidence interval 4.7–10.2%).

The size of the noncompliers group only allowed us to do a descriptive analysis of comparisons between the two groups due to the very low power of the tests. The age in the adherent group was slightly older, 57.8 ± 9.7 years old compared to the mean age of the nonadherent group of 46.9 ± 18.7 years old, *P*  value = .075. The average AHI of the adherent group was 44.9 ± 25.4 versus 36.3 ± 12.3 in the nonadherent group with a *P*-value of  .517. Finally, 40% of the adherent groups were females compared to 25% of the nonadherent group. The difference was not statistically significant with a *P*-value of  .603. 

A second analysis was performed on the group of compliers defined just by considering the 1-year or longer visit regardless of whether or not they had demonstrated compliance at the first visit. Of the original 368 patients who received CPAP machines, 95 subjects followedup at 1 year. Forty-six patients were using their machines at one year while 49 were not (Figure 2). This makes the compliance rate 12.5% (95% confidence interval 9.3–16.3%). Considered singly, none of the variables, gender, race, AHI, or age, showed significant differences between the two groups. Examining the variables individually identified one significant relationship. The shorter the length between the initial sleep visit and receiving the CPAP machine was associated with improved compliance (*P* = .014) (Table 1).

Checking the demographic variables with a multiple variate logistic model including two-way interactions showed significant age by AHI (*P*-value = .033) and age by female (*P*-value = .085) interactions. We then fit stratified multiple variate models where we stratified by sex. The multiple variate logistic regression applied separately to males and females showed a significant AHI variable (*P* = −.026) and a significant AHI by age interaction (*P* = .023) for males. The modeling for females showed no significant terms (Table 2). The coefficients indicated that for males with AHI greater than 27.3, older subjects are more likely to be compliant. Setting AHI at 45.8, a 10-year increase in age increases the likelihood of being compliant 1.74 times. For AHI, if age is greater than 52.7, higher values of AHI are associated with a higher likelihood of compliance. At the age of 55.4, a 10-unit increase in AHI is associated with an odds ratio of 1.09 which goes up to 1.25 at the age of 60 years.

As a final analysis, we also considered the socioeconomic variables and the various log-transformed time variables to examine their impact on compliance at 1 year. Utilizing a stepwise logistic regression strategy, we identified 4 variables in a model, which all significantly predicted 1-year compliance. The two time variables, from the first sleep appointment to the polysomnogram and the total time until initiation of CPAP therapy, the percentage of the population in the neighborhood of the subject who had not finished high school, and the median household incomes were all significantly related to compliance. A higher likelihood of compliance was associated with a longer time from sleep appointment to polysomnogram, a shorter total time, a higher percentage of non-high-school graduates, and higher incomes (Table 3).

## 4. Discussion

Five hours was chosen as the number of hours per night to be considered compliant based on data that suggest that >4-5 hours of CPAP usage per night results in improvement in Epworth Sleepiness Scale scores. [3, 4] Stepnowsky and Dimsdale demonstrated that higher rates of compliance (i.e., >4 hours of usage per night) resulted in an improvement in the respiratory disturbance index, oxygen desaturation index, and arousal index. [5] Campos-Rodriguez et al. demonstrated improved survival rates in those patients that used their CPAP machines greater than 6 hours per night and between 1 and 6 hours per night, as opposed to less than 1 hour per night [6].

It has been shown that optimal adherence to CPAP can be predicted as early as the third to seventh day of usage. Budhiraja et al. [7] demonstrated that 86% of their patients who used their machines for greater than 4 hours a night on day 3 were still using greater than 4 hours a night at day 30. Popescu et al. looked at patients with adherence at 2 weeks and one year. Seventy three percent of the patients elected to continue their CPAP after a 2-week trial. [8]Of those patients that continued to use the machine for a longer duration, 68.5% were still using them for greater than 5 hours per night at one year. [8] Our study looked at the 1-to 4-month interval usage as being a predictor of long-term usage. Only 26 of 106 patients (24%) were noted to remain adherent to CPAP at one year if they were adherent at one month which seems low compared to the other studies.

Others have looked at increasing age as a predictor of adherence to the CPAP machines. [7, 9, 10] The average age in the adherent group was 57.8 years old compared to the nonadherent group of 46.9 years old although this was not statistically significant. At the age of 55, a 10-unit increase in AHI is associated with an odds ratio of 1.09 which goes up to 1.25 at the age of 60 years (*P* = .023). The reason that older individuals are more compliant may be secondary to the associated stigma of CPAP machine usage in the younger generation. Perhaps younger individuals are not as well educated about OSA or do not grasp the consequences of failure of use. 

Twenty four point three percent of our patients were noted to be female which is a consistent percentage compared to other studies by Budhiraja et al. 22% and Sin et al. 18.9%. Gender appears to be a predictor of adherence in our study when one looks at it in a multivariate analysis; older males had a higher tendency to adhere to CPAP usage. This finding is contrary to Budhiraja et al. and Sin et al. who did not find any gender predilection for adherence. 

AHI was examined to determine if it was a predictor of compliance. The average AHI in the population that consistently used CPAP was 44.9, and the AHI in the group that was less consistent or did not use CPAP was 36.3. This was not, however, statistically significant. This is consistent with Yetkin et al. who noted that of three groups of patients, divided on the basis of their AHI scores, those with severe sleep apnea (mean AHI 56.6 ± 27.7) had a greater tendency to utilize CPAP regularly [11]. 

Socioeconomic class can contribute to patient adherence to CPAP. It has been shown that those of low socioeconomic class have less of a tendency to be compliant. Simon-Tuval et al. looked at 162 patients and ranked patients according to their income levels as below (<20%), equal to (±20%), or above (>20%) the average national monthly income level. They found that 40% of all of the patients were adherent to CPAP, and for each increase in income level category, the odds for CPAP acceptance increased by 140% (95% CI). In our study [9], a higher likelihood of compliance was associated with higher incomes which is consistent with Simon-Tuval et al. 

It is of interest that those without high school educations are more likely to be compliant with CPAP. Nino-Murcia G et al. also showed that patients with a high school education or less were statistically significant more compliant than those with higher levels of education [12]. This significance is not clear and may be because they take medical advice at face value and are less likely to question therapy.

As the time between the initial office visit and initiating CPAP therapy is increased, the chance that a patient will be compliant will decrease. The time from the initial visit at a sleep center to receiving the CPAP machine was a geometric mean time of 85.9 days. The geometric mean time from the polysomnogram to CPAP titration was a mean of 35.4 days. The time between the initial polysomnogram and CPAP titration was longer for non-African American females with higher incomes. There has been research looking at split night sleep studies to determine if the reduction in time from initial polysomnogram to time of receiving the CPAP machine overall may ultimately improve compliance. Collen J et al. looked at this question and examined 267 patients that underwent a split night study and 133 patients that underwent a dual night study. The mean number of days between their diagnosis and titration was 80.5 days in comparison to our actual mean of 52.9 days. There was no difference between groups in terms of percentage of nights CPAP was used (78.7% versus 77.5%; *P* = .42), hours per night CPAP was used (3.9 versus 3.9; *P* = .95), or percentage of patients using continuous positive airway pressure for >4 hours per night for >70% of nights (52.9% versus 51.8%; *P* = .81) [13]. Sanders et al. also showed that there was no difference with respect to adherence between their split night group who used 5.1+/−4 hours of CPAP per night in comparison to the two night groups who used their CPAP 4.6 ± 3 hours per night (*P* = .64) [14]. 

## 5. Conclusion

CPAP is the treatment of choice for obstructive sleep apnea if there is adequate patient compliance which raises the issue of efficacy and effectiveness. However, as in other studies, compliance continues to be an issue. Older males with higher AHI values were associated with a better compliance as were patients with higher income levels. Interventions to improve CPAP interventions need to be targeted at young males and females to improve adherence. 

There is also a significant gap in care for patients that undergo polysomnogram with a resultant diagnosis of sleep apnea but fail to obtain CPAP therapy. Length of time from initial visit for complaints of sleep apnea and initiation of therapy is quite long. In the current system, multiple visits for diagnosis and treatment are necessary which may impact the engagement of patients with subsequent CPAP therapy. Efforts should be made to try to minimize the length of time between the initial visit and the patient receiving their CPAP machine to improve compliance. Decreasing the time between the initial polysomnogram and CPAP titration was associated with better compliance.

##  Conflict of Interests

The authors declare no Conflict of Interests.

## Figures and Tables

**Table:** (a)

	*Non compliers*			*Compliers*			
Variable	*n*	mean	SD	*n*	mean	SD	*P*-value
Age	49	54.2	13.4	46	56.7	10.3	.306
AHI	49	44.77	29.7	46	46.9	24.4	.697
ln time from initial visit to initial polysomnogram	46	3.19	1.25	43	3.22	0.77	.887
ln Time from initial polysomnogram to CPAP titration	47	3.73	0.79	46	3.53	0.71	.206
ln Time from CPAP titration to receiving machine	47	2.98	0.76	44	2.72	0.46	.050
ln Total time from initial appointment to receiving CPAP machine	44	4.64	0.71	41	4.28	0.60	**.014**
Median household income	47	$52,670	$25,060	46	$62,280	$29,030	.091
Household size	47	2.45	0.64	46	2.65	0.41	.077
Household size owners	47	2.58	0.66	46	2.69	0.43	.315
Household size renters	47	2.15	1.28	46	2.49	1.16	.180
ln percent less than high school education	46	2.54	0.80	46	2.56	0.76	.919
ln percent high school education	46	28.56	10.06	46	26.49	10.01	.325
ln percent college education	46	55.91	18.38	46	57.90	18.32	.605

**Table:** (b)

	Noncompliers	Compliers	
	% (*r*/*n*)	% (*r*/*n*)	*P*-value

Female	34.7 (17/43)	26.1 (12/46)	.363
Black	37.0 (17/46)	26.7 (12/45)	.292

**Table 2:** The patients were stratified by sex and placed into a logistic regression model predicting group. The model for females showed that no variables were significant. The model for males, however, had a significant AHI variable (*P*  value = .026) and a significant two-way interaction between age and AHI (*P*  value = .023). Older ages with higher AHI values are associated with a higher chance of being compliant. There were sixty-six observations with 32 compliers that were used in the analysis for males and 29 observations with 12 compliers that were used for the analysis of females.

Sex	Variable	Coefficient	*P*-value
Male	Age/10	−0.83	.148
	AHI/10	−1.58	**.026**
	Inter	0.3	**.023**

Female	Age/10	−0.57	.612
	AHI/10	0.23	.877
	Inter	−0.01	.975

**Table 3:** Results of backwards logistic regression predicting 1-year compliance. Eighty-three observations with 41 compliers used in this analysis.

Variable	OR (95% CI)	Coefficient	*P*-value
ln percentage of the population without a high school degree	3.05 (1.03, 9.03)	1.115	**.044**
ln time from initial sleep appointment to initial polysomnogram	2.82 (1.14, 6.93)	1.035	**.024**
ln total time from initial sleep appointment to receiving CPAP machine	0.11 (0.03, 0.44)	−2.247	**.002**
Median household income	1.04 (1.01, 1.08)	0.043	**.010**
